# Optimizing SIEM Throughput on the Cloud Using Parallelization

**DOI:** 10.1371/journal.pone.0162746

**Published:** 2016-11-16

**Authors:** Masoom Alam, Asif Ihsan, Muazzam A. Khan, Qaisar Javaid, Abid Khan, Jawad Manzoor, Adnan Akhundzada, M Khurram Khan, Sajid Farooq

**Affiliations:** 1 Dept. of Computer Science, COMSATS Institute of Information technology, Islamabad, Pakistan; 2 Trillium Information Security Systems, Rawalpindi, Pakistan; 3 NUST College of EME, National University of Sciences and Technology, Islamabad, Pakistan; 4 Deptt of Computer Science, International Islamic University, Islamabad, Pakistan; 5 King Saud University, Riyadh, Saudi Arabia; West Virginia University, UNITED STATES

## Abstract

Processing large amounts of data in real time for identifying security issues pose several performance challenges, especially when hardware infrastructure is limited. Managed Security Service Providers (MSSP), mostly hosting their applications on the Cloud, receive events at a very high rate that varies from a few hundred to a couple of thousand events per second (EPS). It is critical to process this data efficiently, so that attacks could be identified quickly and necessary response could be initiated. This paper evaluates the performance of a security framework OSTROM built on the Esper complex event processing (CEP) engine under a parallel and non-parallel computational framework. We explain three architectures under which Esper can be used to process events. We investigated the effect on throughput, memory and CPU usage in each configuration setting. The results indicate that the performance of the engine is limited by the number of events coming in rather than the queries being processed. The architecture where 1/4th of the total events are submitted to each instance and all the queries are processed by all the units shows best results in terms of throughput, memory and CPU usage.

## 1 Introduction

Over the last decade, with the increase in computer applications and services, the need for processing larger quantities of data has also increased. Data is generated from various sources, including social networks and media, mobile devices, internet transactions and networked devices and sensors. This enormous increase to process large quantities of information introduces scalability challenges in large distributed systems. This sets the scene for the era of “big data” [1]. In context, Facebook has 890 million daily active users and 1.39 billion monthly active users on average as of December 31, 2014 according to reports on the Facebook Newsroom [2]. More than 5 billion people are calling, texting, tweeting and browsing via mobile phones worldwide. Akamai analyzes 75 million events daily to improve targeting advertisements. Walmart handles more than 1 million customer transactions every hour. Appliances such as sensors are widely deployed both in daily life and research for monitoring the environment. In networks, we have a variety of sensors such as thermal, visual, acoustic and radar. These sensors can monitor ambient conditions, including temperature, humidity, pressure, noise level, movement of certain objects [3]. Sensors are also producing large amounts of data continuously over time. The large volume of data generated in all manners challenges existing information technology (IT) architectures to process information effectively and efficiently. Cloud computing is a new IT paradigm which has gained tremendous popularity. In this model resources like bandwidth, storage, servers, processing power, services, and applications are pooled and given to the end user in pay as you go manner. Essential characteristics of the cloud-computing model include on-demand self-service, rapid elasticity, resource pooling and broad network access. This paradigm has eliminated the overhead of planning from the user, by providing resources that are available on-demand, self-service, and the ability to scale according to user requirements [4]. Cloud computing has been applied in many diverse domains [5] [6] [7] [8]. The analysis of large volumes of heterogeneous data easily becomes a performance bottleneck for applications. Much research has gone into progressive work on the various aspects of the challenges of big data [9] [10] [11] [12] [13]. The focus of our work specifically is the analysis of Security Information and Event Management (SIEM) [14]. SIEM systems perform real time correlation of the heterogeneous data collected from different sources and report threats, communication with botnets [15] [16] [17] [18], monitor business processes [19] [20], anomalous patterns [20] and, other kind of attacks [21] [22] in the organizational data. For real time correlation, SIEM makes use of complex event processing (CEP) systems. Complex Event Processing (CEP) [23] extracts meaning out of the huge amount of data. In security and mission critical systems, real time processing of the data is very important. The performance of such systems must not degrade with the increase in volume of data. A SIEM solution can be deployed in a particular organization for providing a unified view of the overall network activity taking place. However, deploying and managing a SIEM solution is expensive and needs dedicated and skilled human resources. The general scarcity of required skill set often results in a half-cooked SIEM deployment, which provides a false sense of security. The understanding of issues related to security solutions deployment has given rise to another business model, where security is being offered as a service, a concept called Managed Security Service Provider (MSSP) [24]. Companies outsource part of their security monitoring to the MSSP. The MSSP mostly hosts their SIEM solution using cloud computing. Multiple clients are registered with MSSP and the event data is fetched from the client network to the SIEM. SIEM analysis the event data using security rules and reports malicious and anomalous activities. However, as the clients of an MSSP increase, the events per second increase exponentially, which reduces the capacity of real time processing of these events. The events data starts accumulating in the queues and once the queuing capacity is overloaded, the events data starts getting lost. There is a need to offer innovative ways of data processing in a managed SIEM solution that can handle several thousand events per second without compromising the real time processing ability. Our research group, with the help of an industrial partner, has launched the services of an MSSP in Pakistan [25]. The ensuing research work stems out of the actual challenges that we have faced while offering SIEM as a managed service. As MSSP we offer ‘SIEM as a Service’ at Client Site and ‘SIEM as a Service’ in the Cloud. For either option, it is important to make best use of the computing resources for providing service as per the service level agreement (SLA) and to keep the costs low. For the latter option, performance becomes an even bigger issue as the events per second increase significantly with the addition of more clients. Before we could offer SIEM as a service in the Cloud, it was important to thoroughly test the capability of our system. In our experimental setup (discussed in section 4), the received events data are fed to the correlation engine of our SIEM framework called OSTROM. The queries of our SIEM security rules contain large time windows, complex aggregators and custom function calls. We have currently 148 security rules, which are executed in the correlation engine. Under normal circumstances the performance of the correlation engine is slow and can process a maximum of 44,000 events with 1,000 events per second. Even though Esper claims to be a parallel engine and has a benchmark [26] published with 500,000 events per second, in our case we realized that it is not very efficient in parallelizing rules. Esper, although claimed to be inherently parallel, cannot make a suitable parallelization decision when overwhelmed with a large quantity of event data. Hence, a manual parallelization of event data is recommended. In order to test this hypothesis and to improve the capability of our SEIM solution significantly, we conducted an experiment using three different processing architectures. Our experimental results support our hypothesis that the architecture that parallelizes queries is the most optimal solution and can help MSSPs while providing security services to their clients. Objective of the paper is to enhance the performance of the TRIAM RDSA SIEM. Currently it is able to handle to 250 events per second (EPS). This EPS is very low to compete in the market of the SIEM. Our target is to achieve 5000 EPS by finding out the bottleneck in the system and removing it.

Our contribution in this paper are the followings:

- This paper evaluates the performance of a security framework OSTROM built on the Esper complex event processing (CEP) engine under a parallel and non-parallel computational framework.
- We propose three architectures under which Esper can be used to process events. We investigated the effect on throughput, memory and CPU usage in each configuration setting. The results indicate that the performance of the engine is limited by the number of events coming in rather than the queries being processed.
- In this paper we have achieved 5000 EPS which is one of major milestones. During struggle to 5000 EPS achievement we have also analyzed the performance of Esper CEP engine used in correlation engine and concluded that Queries complexity has higher impact on the CEP engine performance.

*Organization of the paper*: This paper is organized as follows: Section 2 describes the related work on this problem, in section 3 proposed system architecture is presented. Section 4 describes experimental evaluation and discussion on the results. In section 5 conclusion is presented.

## 2 Related Work

Miyuru et. al [27] compared the performance characteristics of EsperTech’s Esper with IBM S and Yahoo’s S4 System in a distributed environment. Miyuru used a simple micro benchmark and three applications repeatedly doing the same activity, which is not effective in system/solutions like SIEM, where the detection of different kinds of patterns and activities is required all at the same time. Driver queries detect activities where these queries have different windows sizes (both time and length-wise) and some detect patterns through Esper pattern queries. Kuboi et al. [28] calculated throughput by increasing the input, window size for pre-defined “sum”, “count”, “avg”, “min”, “max”, “stddev”, “median”, and “avedev” algorithms and by increasing the length of the patterns. Again, their experiments were limited to the selected algorithms and two kinds of co-occurrence followed by patterns. One limitation of this work is that, it did not investigate the effect of various queries on the performance of the system. Grabs et al. [29] also worked on the performance testing of the Esper in an automated environment. They tested Esper for latency, execute pollution test, and load testing and all this was in the virtualized environment using Oracle VirtualBox. However, this work failed to identify significant differences in the results of the latency test applying load to the test candidates. The authors intended to reinvestigate load tests in more detail in their future work. Again our work is different from them in number of queries and diversity in queries. EsperTech has published its own benchmarking results for Esper, using simple queries only of different size windows of time and length. We are looking at its performance in a case when it will be integrated with the SIEM for real world diversified activity detection use case. In a further publication, Mendes et al. [30] use their framework to perform different performance tests on three CEP engines—Esper and two developer versions of not further specified commercial products. They run “micro benchmarks” while they vary query parameters like window size, windows expiration type, predicate selectivity, and data values. So, they focus on the impact of variations of CEP rules. Beside they perform some kind of load test. The results thereby showed a similar behavior in memory consumption like Esper did in our tests. In summary, Mendes et al. did run performance tests, but again our setup is with different kind of complex queries with custom function calls. White et al. [31] published a performance study for the WebLogic event server which is now known as now Oracle CEP. In that work, solely latency testing is performed on a single CEP system. But in our work we are testing the system (SIEM solution) by creating multiple CEP instances. Ku Mu et al. [32] observed the performance effects of these performance parameters, by implementing and running an Esper-based event processing application on top of a Xen-based virtualized system. They analyzed the memory consumption problem, and apply periodic garbage collection, to reduce unexpected memory consumption of JVM. Also, analyzed performance effects of the number of cores in a virtual machine (VM) and resource sharing among VMs. Coppolino et al. [34] also discussed coordinated and targeted cyber attacks on critical infrastructures. Furthermore, the authors have also talked about the limitations of SIEM systems and provided a prototype deployment for the dam monitoring. Many other CEP solutions [36], among which BEA WebLogic [37] now used in Oracle CEP, has also been using Esper CEP engine, every solution is using it in their own way and obtained results according to their environment settings. Here we are using it in our SIEM solution, where we have different results when single Esper engine with heavy load settings and multiple Esper engine with load distributed among engines setting in configured. A number of survey articles have also reviewed complex event stream processing such as [33] and [35]. Wrench et al. [35] recently surveyed techniques of data stream mining of event and complex event streams. The authors argued that volume, velocity, veracity and variety characteristics of big data has to be accounted for while mining events and complex event streams as these characteristics demand special consideration. Cammert et al. [38] technique can forecast the future behavior of event streams. This scheme is quite useful in predicting future behaviors in complex event processing environment. However, the scheme is a patent. Sejdovic et al. [39] proposed a proactive disruption management system for manufacturing environments. This system helps being prepared for the unexpected by applying a combination of unsupervised and supervised machine learning for the identification and prediction of unspecified situations. Furthermore, it adopts data mining techniques to derive predictive patterns for specified situations. The authors have also introduced a real-world use case from the field of semiconductor manufacturing and presented their preliminary results showing how possible disruptions and how they can be detected from the sensor data. Rieke et al. [40] proposed an approach to support evaluation of the security status of processes at runtime, which is based on operational formal models derived from process specifications and security policies comprising technical, organizational, regulatory and cross-layer aspects. The scheme synchronizes a process behavior model from the running process and utilizes prediction of expected close-future states to find possible security violations and allow early decisions on countermeasures. Using a case study of hydroelectric power plant, a misuse case scenario is described. Rieke et al. [41] proposed scheme supports security compliance tracking of processes in networked cooperating system. The scheme proposed is an advanced method of predictive security analysis in runtime using established methods in data mining, machine learning, predictive analytics, complex event processing and process mining. The proposed scheme is demonstrated by applying it in an industrial application of critical infrastructure process control. Gad, Rüdiger [42] improved the state of the art of network analysis and surveillance. The work aims towards holistic network analysis and surveillance by addressing distribution, convergence usability and performance aspects. It showed the benefits and evaluate the applicability of event driven data processing paradigms. Furthermore, it also showed how self-adaptability and cooperation can further improve the capabilities.

Our work is different in the sense that we are working with a variable number of queries with many different kinds. Our queries range from simplistic queries which use filters only to more complex. We have queries that use aggregate functions along with a single row custom function call while some use on demand query execution inside the custom functions. Others still compare input events to the stored data and we also have queries detecting different patterns. Such a varied mixture of queries affects the performance of the Esper-based system. We investigate how performance, CPU and memory usage of the system is affected by increasing/decreasing the Esper engine instances and reducing the number of queries and/or numbers of input events to the Esper Complex event processing engine.

**Algorithm 1** Architecture 1

0: **procedure** Architecture1(**event** *n*, **rules** *m*)

1: **for** (*i* = 0; *i* ≤ *n*; *i* ++) **do**

2:  PROCESSEVENTt(n,m)

3: **end for**

## 3 Proposed System Architecture

In this research work we have taken a cloud based Managed Security Service Provider (MSSP) as our use case. In a cloud based MSSP, we have mainly looked at SIEM as a Service deployed over the cloud. Clients are registered with the MSSP and the events data of their hardware and applications are brought to the SIEM. Logs are preprocessed and then correlated by the SIEM and results are stored in the database. These results are visible through a web framework to corresponding clients. Generally, as the incoming data volume increases, system performance degrades proportionally. As a solution, the Hadoop MapReduce [43] architecture can be put in place but it does not provide real time data analytics. For near real time security analytics, Storm [44], a parallel real-time computation system, is used for parallel processing of the incoming streams. We have used Apache Storm for stream processing and Esper EPL [45] for complex event processing queries. We have written SIEM rules for common network attacks and have benchmarked their performance. We have introduced explicit parallelization of rules to improve overall performance of the system.

**Algorithm 2** Event Processing by Policy Engine

0: **Procedure** ProcessEvent(**event** *n*, **rule** *m*)

  **for** (*i* = 0; *i* ≤ *m*; *i* ++) **do**

2:  **if** (event[i] satisfies all rule[i] predicate]) **then**

   **if** (event[i].type = “stateless”) **then**

4:    rule[I] is fired

     Enqueue(Event[i])

6:    **end if**

  **end if**

8:  If(event[i].type = “statefull”)

  **For**(*j* = 0; *j* < *timeCount*; *j* ++)

10: Enqueue(Event[i])

   **if** Time bundle rule condition is satisfied **then**

12:   rule[i] is fired

    Output all events

14:  destroy all instances

    **if** Time bundle rule condition is not satisfied **then**

16:   DEQUEUE(event[i])

    **end if**

18:  **end if**

  **end for**

  **end procedure = 0**

**Algorithm 3** Architecture 2

0: **procedure** Architecture2(**event** *n*, **rules** *m*)

 **for** (*i* = 0; *i* ≤ *n*; *i* ++) **do**

  PROCESSEVENTt(n/4,m)

3:  PROCESSEVENTt(2n/4,m)

  PROCESSEVENTt(3n/4n,m)

  PROCESSEVENTt(4n/4,m)

6: **end for**

**Algorithm 4** Architecture 3

0: **procedure** Architecture3(**event** *n*, **rules** *m*)

 **for** (*i* = 0; *i* ≤ *n*; *i* ++) **do**

  PROCESSEVENTt(n,m/4)

  PROCESSEVENTt(n,2m/4)

4:  PROCESSEVENTt(n,3m/4)

  PROCESSEVENTt(n,4m/4)

 **end for**

**Fig 1:**
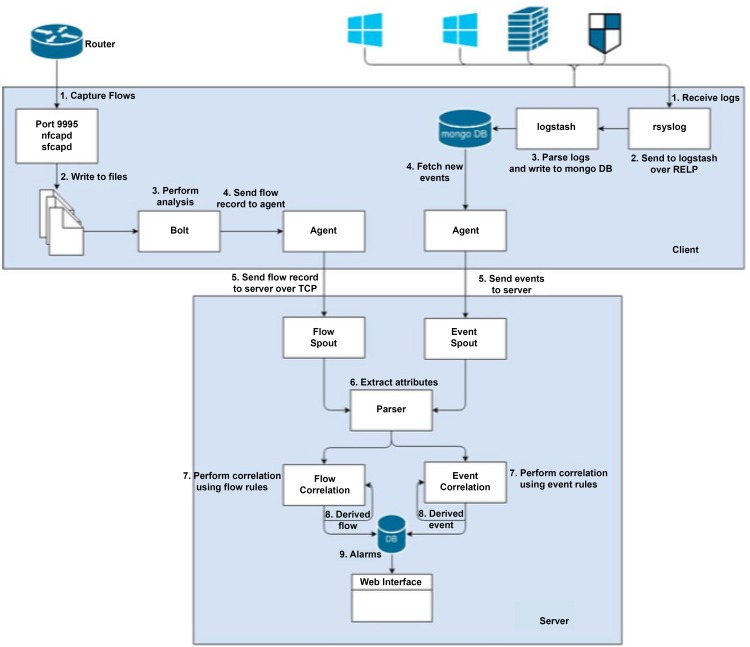
OSTROM Architecture.

Apache Storm has been selected as it is an open source reliable distributed real time computation system. The overall architecture of OSTROM is shown in Fig 1. We have a spout listening on a specific port and is continuously collecting data from different sources. The spout is connected to the parser bolt and passes event data stream to parser bolt which preprocess it. During pre-processing, the data stream is parsed, thus breaking down the event data into required attributes. In the correlation engine, the attributes extracted from the events data is matched against the rules on the fly. Alarms are generated as soon as any malicious or unwanted activity is detected. These results are then input into the bolt, called the DB Writer bolt, which writes the results to the database. As the number of events increase at the correlation bolt, the processing capability degrades, events data begins to accumulate in the queue, and eventually the parser and spout performance degrades and finally comes to a halt.

The most important component of the SIEM is its correlation engine and analysis facility. It correlates the events data to the security rules. For getting real-time results, the rules should be well-written and efficiently executed. The data correlation can be done sequentially or in parallel. Assuming we have 10 security rules and a single event stream then the first approach is to match a single event to rule 1 to n sequentially. If the rule matching of a single event to a single rule takes 1 millisecond, then matching a single event to all 10 rules will take 10 milliseconds. The second approach follows the parallel processing of events that matches a single event to all 10 rules at the same time and hence take 1 millisecond in total.

For events correlation and complex event processing we have used the Esper platform which is already parallelized and has a benchmark published with 500,000 events per second [46]. OSTROM uses Event Processing Language (EPL), one of the SQL based event processing platforms for real time processing. Other SQL based event processing platforms includes Oracle OEP [47], Streambase [48] [49] and Sybase ESP (formerly Aleri) [46]. In Esper, live events are matched against stored rules. We tested Esper in OSTROM by placing simple queries used in the Esper benchmark. The Esper benchmark provides 17 queries by default; in order to stress test the system we increased the number of queries to 300 by replication. The test validated the performance claims of EsperTech in its own benchmark. However, when we placed our 148 real-world attack and anomaly detection rules in Esper then it barely managed to process 44,000 events at the rate of 1000 eps. The difference can be attributed to the fact that the Esper benchmark rules used were simple and were not tested with single-row functions. When we converted our own queries into Esper queries, they became complex.

## 4 Implementation and Evaluation

To test our hypothesis and resolve the performance issues mentioned in section 1, we created an experiment. We had two setups, one involved running a single Esper instance and the other had 4 Esper instances running concurrently. These setups were used to test the throughput capability under three different processing architectures: One sequential (the control) and two parallel.

**Fig 2:**
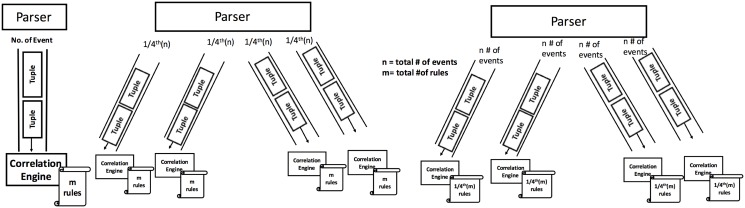
The 3 processing architectures.

The first architecture called Arch-1 involved submitting all the queries to a single bolt on a single Esper instance and assuming Esper will manage parallelization. In the second architecture called Arch-2, we replicated all the 148 queries on each Esper instance whereas 1/4th of the events were submitted to each Esper instance. In the third architecture we used 4 Esper instances and submitted 1/4th of the queries to each instance. Each event was replicated to all four Esper instances. The 3 processing architectures are shown in Fig 2

For measuring CPU and memory usage we used the Java Monitoring and Management Console which is part of CentOS, a variant of the Linux operating system. Each processing architecture was fed events data at the rate of 1000 Events per Second (EPS) with a total of 44 batches of events. We then calculated the time taken by the Esper to execute each batch. For calculating the throughput of OSTROM we developed a separate module which takes the difference between the system time taken when the 1st event was submitted to the engine and the time when the last (1000th) event is successfully executed by the Esper. The tests were performed using a a total of 4 processing cores, 32 GB of RAM, and a storage space 4 TB, running 64 bit CentOS 6.5.

### 4.1 Architecture 1

**Fig 3:**
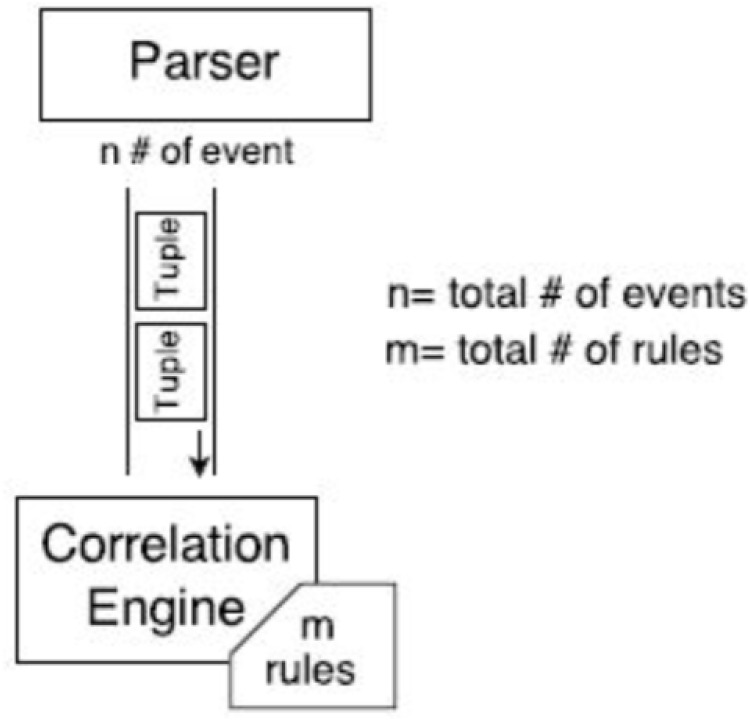
Arch-1: Events processing using a single CEP instance.

Arch-1 consists of a single CEP instance as shown in Fig 3. The results showed that on average it took 3 seconds to execute 1000 events. However, due to the variable complexity of the queries, we noticed a spike in processing activity at the 29th and 38th batches respectively. Executing the 29th batch took 25 seconds, while the 38th batch consumed 71 minutes, significantly skewing the results. Due to this effect, the overall time taken by the CEP instance to execute 44,000 events was 77 minutes which is fairly large amount of time.

**Fig 4:**
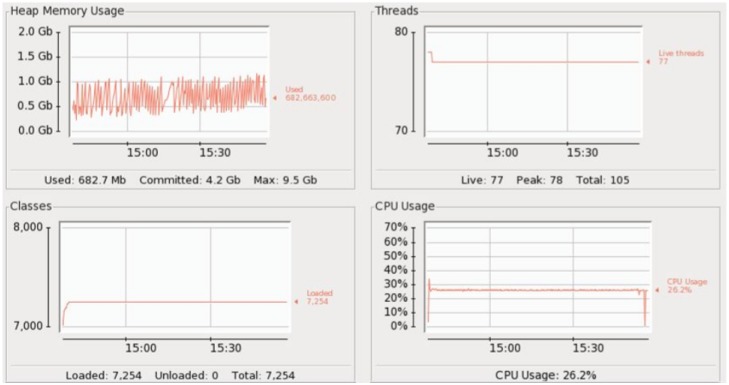
Arch-1: Events processing using a single CEP instance.

CPU and memory usage is shown in Fig 4. Here we see that the CPU usage is the same 30% throughout the events execution which shows that Esper did not efficiently utilize the CPU. Heap memory usage is highly irregular while it gradually increases as we are keeping all those events in memory which fulfill the query predicate.

### 4.2 Architecture 2

**Fig 5:**
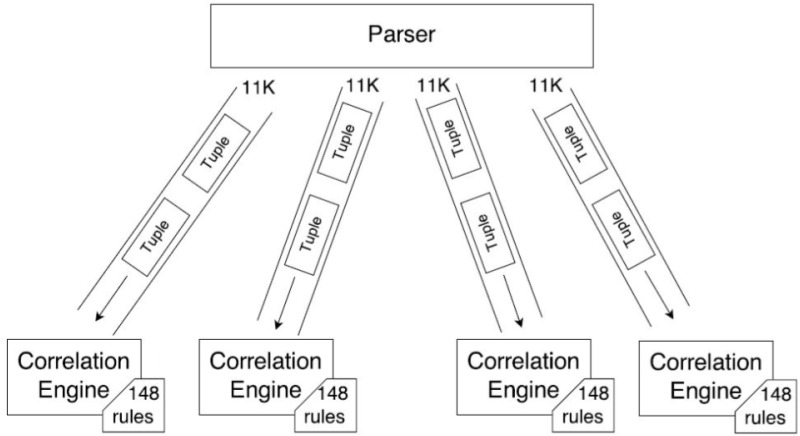
Arch2: 4 CEP instances with the same number of rules while events data is equally distributed.

In Arch-2 we deployed 4 CEP instances (i1, i2, i3, i4) with 148 rules as shown in Fig 5, but this time each instance was given 1/4th of the 44,000 events. We reduced the total number of events submitted to each Asper instance while keeping the same number of rules. We found that each instance ran smoothly, taking 3 seconds to execute each 1000 events batch. No delay was observed in executing each batch of events, therefore all of the 4 CEP instances (i1, i2, i3, i4) consumed 33 seconds each to execute all of their batches.

**Fig 6:**
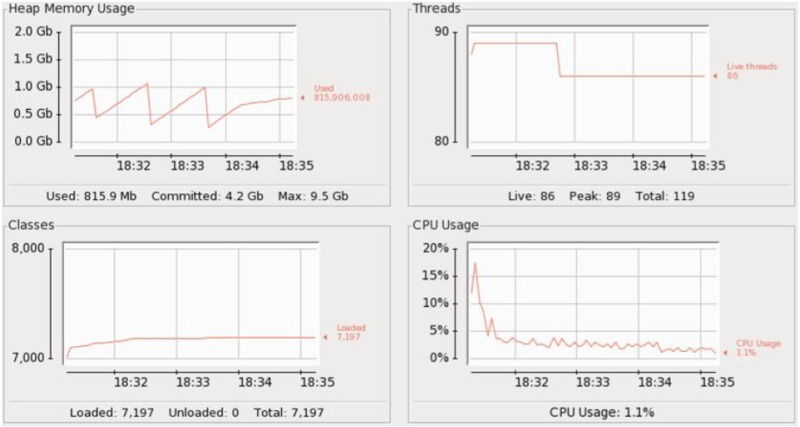
CPU and Memory usage for Arch2.

Fig 6 shows statistics for the Arch-2. It shows less CPU usage as compared to the Arch-1. Memory usage is almost the same as that of the Arch-1. The major drawback of Arch-2 is that event data is not aggregated (i.e. each query duplicate is run separately, unaware of what its other clone is doing), resulting in scattered counts per rule. This adversely affects the triggering of alarms.

The CPU and memory usage is shown in Fig 6. The CPU usage starts at 11%, quickly peaking at 17% and gradually descending to 1.1% thereafter. Here we see that due to event data parallelization the CPU remains idle for most of its time. Memory usage is more consistent and evenly spaced compared to Arch-1.

### 4.3 Architecture 3

**Fig 7:**
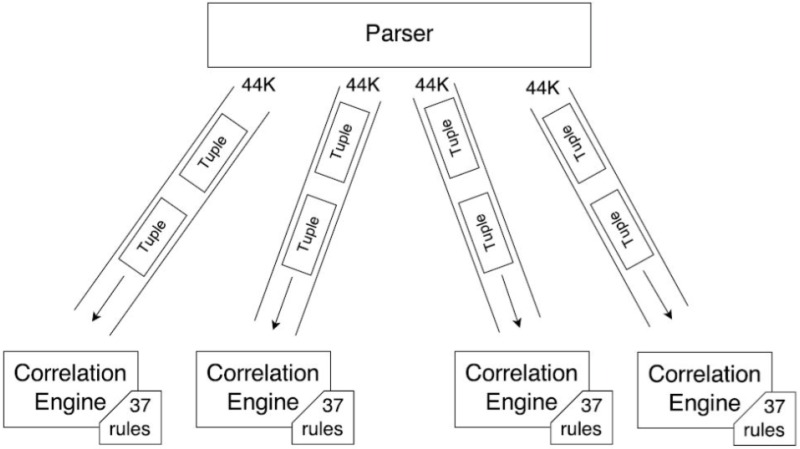
Multiple (4) CEP instances with rules equally distributed over each instance and same number of events are given to instance.

**Fig 8:**
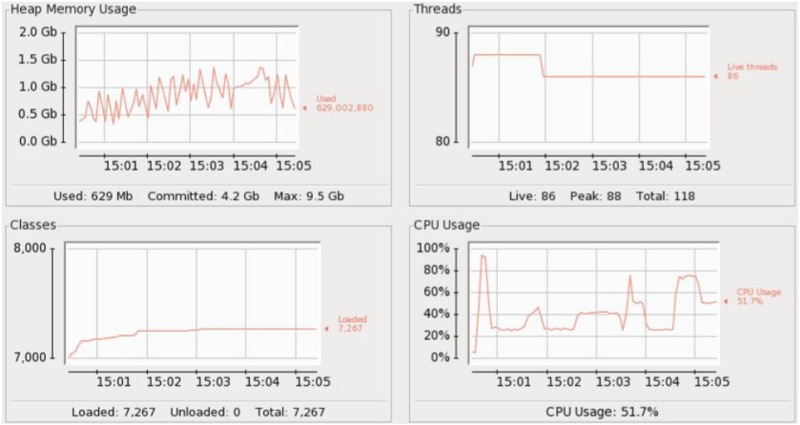
Arch-3: Multiple (4) CEP instances with rules equally distributed over each instance and same number of events are given to each instance.

In Arch-3, we used the same 4 CEP instances (i1, i2, i3, i4), however, we swapped the role of events and queries compared to Arch-2. This means that events are sent at full load (i.e. 44,000) while 1/4th of the 148 queries are submitted to each Esper instance as shown in Fig 7. The results in Fig 8 showed that each instance took 3 seconds on average to execute 1000 events and no significant delay was observed. CPU usage peaked at 80% then came down to 20%, finally stabilized at 70%. We can see that compared to Arch-2, the CPU usage is consistently high, while the memory usage pattern is highly irregular. This indicates that parallelizing the rules did not have as much benefit since the Esper instances are overwhelmed by the 44000 events.

These are 3 different architectures and we have explained it separately. The impact of each architecture is different than each other. For example, CPU usage is significant in both Arch-1 and Arch-2 while Arch-3 reflects little CPU activity. The CPU is idle most of the time, far from being saturated. This agrees with our initial hypothesis that Esper, although inherently parallel, cannot make a suitable parallelization decision when overwhelmed with events at its backlog. Esper shows high performance in high volume of events for a large number of rules. Arch-2 showed us that the complexity of the rules has a lower impact than the number of overall events sent to the Esper instance. The detail of each architecture is given below.

Arch-1 consists of a single CEP instance as shown in Fig 3. The results showed that on average it took 3 seconds to execute 1000 events. The CPU and memory usage is shown in Fig 4. Here we see that the CPU usage is the same 30% throughout the events execution which shows that Esper did not efficiently utilize the CPU. Heap memory usage is highly irregular while it gradually increases as we are keeping all those events in memory which fulfill the query predicate. In Arch-2 we have deployed 4 CEP instances (i1, i2, i3, i4) with 148 rules as shown in Fig 5, but this time each instance was given 1/4th of the 44,000 events. We reduced the total number of events submitted to each Asper instance while keeping the same number of rules. No delay was observed in executing each batch of events, therefore all of the 4 CEP instances (i1, i2, i3, i4) consumed 33 seconds each to execute all of their batches. In Arch-3, we have used the same 4 CEP instances (i1, i2, i3, i4), however, we swapped the role of events and queries compared to Arch-2. This means that events are sent at full load (i.e. 44,000) while 1/4th of the 148 queries are submitted to each Esper instance as shown in Fig 7.

### 4.4 Query Optimization

The complexity of the system depends upon the type of queries, whether simple or complex. Queries registered to correlation engine define the function that the system performs on incoming events. Due to the large volume and different types of data and different interests on the input events from users, queries registered to event processing systems can be complex. Query may contain time and length based windows, aggregation functions, custom function calls etc. Such complex queries are resource intensive and hence impact the performance of SIEM. From a performance management perspective, the challenge is to find out how the complexity of queries influences performance, which helps to understand and optimize a SIEM system’s behavior.

On analyzing the complexity of queries, we found that the problem lies the custom function call made from inside the query. To confirm our viewpoint, we first submitted 100 simple queries with the filter as given below. Events with 5000 EPS were sent to RDSA. It was able to handle 5000 EPS for an hour and we found no problem in events correlation.

Select *from stream where event_context = ‘Local to remote’;

Now in the next step we increased the complexity of the query and submitted 100 queries with filters and custom functions call as given below.

Select *from stream where event_context = ‘Local to remote’ AND check_list(‘Botnet’, destination_ip);

Then in the next step we added on-demand query execution inside the parent query as shown below.

Select *from stream where event_context = ‘Local to remote’ AND execute_block(‘Athentication Successful’) AND check_list (‘Botnet’, ‘destination_ip’);

When 100 queries were submitted to the engine and events with 5000 EPS were send to RDSA, it was very slow and consumed 77 minutes in processing 44000 events, which is unacceptable. We found that system slows down when we add on-demand query execution to the RDSA rules. For further detailed investigation we submitted single query with on-demand query execution. We found that on-demand query execution inside the parent query consumed one-third of the second. This was the culprit and hence we removed the demand query execution and replace this query with its alternate as given below. Now it was very speedy in processing events.

**Fig 9:**
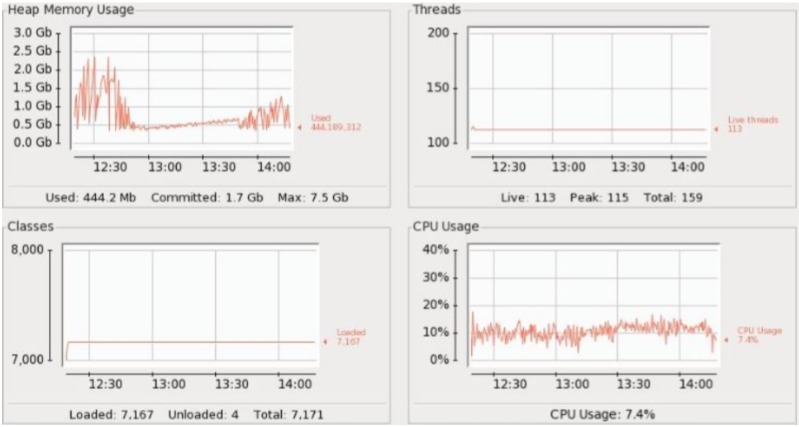
CPU Usage and RDSA statistics in single CEP instance under 500 EPS.

**Fig 10:**
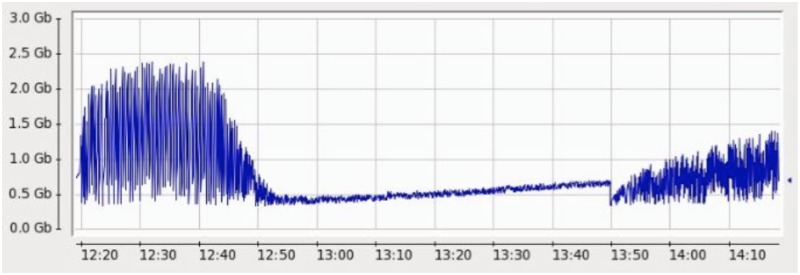
Heap Memory Usage in single CEP instance under 500 EPS.

**Fig 11:**
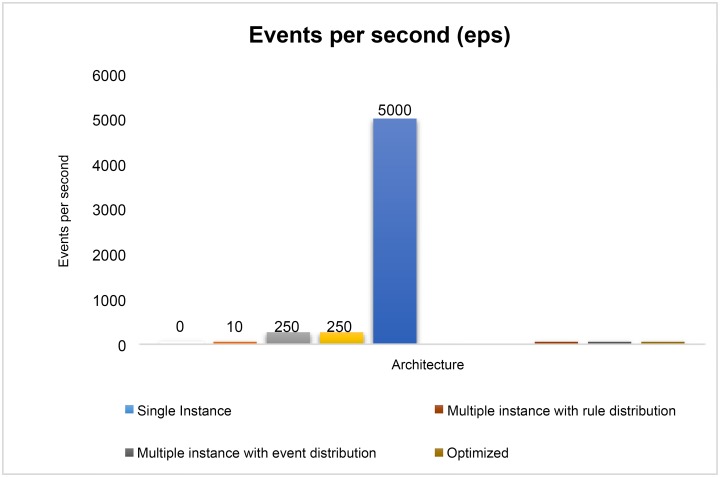
Events Per Seconds Comparison.

Select *from stream where event_context = ‘Local to remote’ AND (category IN (‘Authentication Server’, ‘Admin login Successful’, ‘Host login succeeded’, ‘Mail Server login Succeeded’)) AND check_list (‘Botnet’, ‘destination_ip’)’;

We then replaced all the rules with their alternatives and removed on-demand query execution from inside the query. After re-writing all 148 rules, they were submitted to the engine and now events were sent with 5000 EPS. It was tested for 2 hours and was able to handle events very smoothly. Fig 9 shows the performance of RDSA with optimized queries. Heap Memory usage graph is shown in Fig 10. Fig 11 shows the EPS comparison before and after optimization.

Above graph shows the comparison before and after query optimization. We see that before optimization under single and multiple correlation bolt instances EPS is 10 and 250, which is some improvement in terms of EPS. However, it is good for the small companies with low log generation rate. So we had to move further and improve queries after which we achieved 5000 EPS which is substantial improvement as it can handle medium to large size companies.

## 5 Conclusion

CPU usage is significant in both Arch-1 and Arch-3 while Arch-3 reflects little CPU activity. The CPU is idle most of the time, far from being saturated. This agrees with our initial hypothesis that Esper, although inherently parallel, cannot make a suitable parallelization decision when overwhelmed with events at its backlog. This is apparent in the difference in actual execution times taken all three architectures. Upon explicitly parallelizing events data, we observed a significant reduction in CPU usage, and a reduction in processing time: In effect, a significant improvement in efficiency. The CPU usage went down to 1.1%, whereas the rule processing became real-time, leaving nearly 99% of the CPU still idle for other tasks. We conclude that Esper shows high performance in high volume of events for a large number of rules. Arch-2 showed us that the complexity of the rules has a lower impact than the number of overall events sent to the Esper instance. In such a case, explicit parallelization/batching of events is recommended.
