# Correction: Optimizing SIEM Throughput on the Cloud Using Parallelization

**DOI:** 10.1371/journal.pone.0171581

**Published:** 2017-02-01

**Authors:** Masoom Alam, Asif Ihsan, Muazzam A. Khan, Qaisar Javaid, Abid Khan, Jawad Manzoor, Adnan Akhundzada, Muhammad Khurram Khan, Sajid Farooq

The eighth author’s name is spelled incorrectly. The correct name is: Muhammad Khurram Khan.
